# Assessment of the Cost-Effectiveness and Clinical Outcomes of a Fourth-Generation Synchronous Telehealth Program for the Management of Chronic Cardiovascular Disease

**DOI:** 10.2196/jmir.3346

**Published:** 2014-06-10

**Authors:** Yi-Lwun Ho, Jiun-Yu Yu, Yen-Hung Lin, Ying-Hsien Chen, Ching-Chang Huang, Tse-Pin Hsu, Pao-Yu Chuang, Chi-Sheng Hung, Ming-Fong Chen

**Affiliations:** ^1^Telehealth CenterNational Taiwan University HospitalTaipeiTaiwan; ^2^Department of Internal MedicineNational Taiwan University HospitalTaipeiTaiwan; ^3^National Taiwan UniversityTaipeiTaiwan; ^4^Department of NursingNational Taiwan University HospitalTaipeiTaiwan

**Keywords:** cardiovascular disease, cost-benefit analysis, telehealth

## Abstract

**Background:**

Telehealth programs are a growing field in the care of patients. The evolution of information technology has resulted in telehealth becoming a fourth-generation synchronous program. However, long-term outcomes and cost-effectiveness analysis of fourth-generation telehealth programs have not been reported in patients with chronic cardiovascular diseases.

**Objective:**

We conducted this study to assess the clinical outcomes and cost-effectiveness of a fourth-generation synchronous telehealth program for patients with chronic cardiovascular diseases.

**Methods:**

We retrospectively analyzed 575 patients who had joined a telehealth program and compared them with 1178 patients matched for sex, age, and Charlson comorbidity index. The program included: (1) instant transmission of biometric data, (2) daily telephone interview, and (3) continuous decision-making support. Data on hospitalization, emergency department (ED) visits, and medical costs were collected from the hospital’s database and were adjusted to the follow-up months.

**Results:**

The mean age was 64.5 years (SD 16.0). The mean number of monthly ED visits (mean 0.06 SD 0.13 vs mean 0.09 SD 0.23, *P*<.001), hospitalizations (mean 0.05 SD 0.12 vs mean 0.11 SD 0.21, *P*<.001), length of hospitalization (mean 0.77 days SD 2.78 vs mean 1.4 SD 3.6, *P*<.001), and intensive care unit admissions (mean 0.01 SD 0.07 vs mean 0.036 SD 0.14, *P*<.001) were lower in the telehealth group. The monthly mean costs of ED visits (mean US$20.90 SD 66.60 vs mean US$37.30 SD 126.20, *P*<.001), hospitalizations (mean US$386.30 SD 1424.30 vs mean US$878.20 SD 2697.20, *P*<.001), and all medical costs (mean US$587.60 SD 1497.80 vs mean US$1163.60 SD 3036.60, *P*<.001) were lower in the telehealth group. The intervention costs per patient were US$224.80 per month. Multivariate analyses revealed that age, telehealth care, and Charlson index were the independent factors for ED visits, hospitalizations, and length of hospitalization. A bootstrap method revealed the dominant cost-effectiveness of telehealth care over usual care.

**Conclusions:**

Better cost-effectiveness and clinical outcomes were noted with the use of a fourth-generation synchronous telehealth program in patients with chronic cardiovascular diseases. The intervention costs of this new generation of telehealth program do not increase the total costs for patient care.

## Introduction

Cardiovascular disease (CVD), one of the main chronic diseases, is the leading cause of death and disability worldwide [1]. Chronic CVD is characterized by a high rate of co-morbidities and a high risk for acute deterioration [2], both of which contribute to adverse clinical outcomes and economic burden on society. Hospitalization due to acute deterioration represents the main cost component of CVD care [3]. To reduce hospitalization and improve long-term care for CVD, disease management programs, defined as multidisciplinary approaches that coordinate care strategies to manage patients with chronic disease, have been applied to patients with chronic CVD [4,5]. Despite the beneficial results, disease management programs are limited by their high cost and modest efficiency.

Recently, advances in telemonitoring devices have improved the efficiency and reduced the labor costs of disease management programs. With the help of remote telemonitoring, biometric parameters can be monitored closely and acute episodes of deterioration can be detected early, both of which make timely interventions possible. Telehealth programs, which incorporate telemonitoring and disease management programs, have been shown to improve the results of long-term care in patients with chronic diseases including heart failure, chronic respiratory disease, and diabetes [6-10]. However, not all studies on telehealth programs revealed beneficial results. A recent randomized controlled trial of a telehealth program, which used a non-immediate data analysis system, failed to reduce hospitalizations in elderly patients with chronic diseases (hospitalization and emergency department [ED] visits: telemonitoring group 63.7% vs usual care group 57.3%, *P*=.35) [11]. Patients enrolled in this study were elderly with a mean age of 80.3 years and at high risk for rehospitalization. Another study on a telehealth program among patients with chronic illness including heart failure, diabetes, or chronic obstructive pulmonary disease in the United Kingdom also revealed unfavorable results. The cost-effectiveness study of this telehealth program revealed a slightly higher total cost in the telehealth group (telehealth group, £1596.10 vs usual care group, £1389.70, *P*>.05) but relatively high costs per quality adjusted life year (QALY) gain (£92,000, n=969), and concluded that the telehealth program was not cost effective [12]. A review article published recently also argues that evidence for the benefit of telehealth programs in managing chronic diseases is inadequate and contradictory [13]. These contrasting results raise a serious concern about the use of telehealth programs in the management of patients with chronic CVD.

One possible explanation to these contrasting results is the difference in the level of care provided by telehealth programs. Based on the level of data analysis, decision ability, and integration of care, Anker and coworkers classified telehealth programs into four generations: (1) non-reactive data collection programs, (2) programs with non-immediate analytical structure, (3) remote patient management programs, and (4) fully integrated remote management programs [14]. According to this classification, the fourth-generation telehealth program provides the highest level of patient care: round-the-clock presence of a physician and nursing staff to analyze and respond “synchronously” to the data transferred from patients. It is probable that the different levels of monitoring, staff, and response provided by these four generations of telehealth program contribute directly to the aforementioned contrasting results in the literature. However, the clinical benefit of the fourth-generation telehealth program has not been validated.

Based on these reasons, we hypothesized that a fourth-generation telehealth program would be effective in patients with chronic CVD. To test this hypothesis, we first reported reductions in costs and hospitalization rates for patients with chronic CVD 6 months after vs before receiving a fourth-generation telehealth program in a quasi-experiment study [15]. We then conducted this retrospective cohort study to elucidate whether the patients with chronic CVD who received the fourth-generation telehealth program may have better clinical outcomes and cost-effectiveness compared with those who received standard care in a longer follow-up period.

## Methods

### Study Design

This was a single center, retrospective study, and was approved by the Institutional Review Board at National Taiwan University Hospital, Taipei, Taiwan.

### Recruitment

The study was conducted from December 2009 to April 2013 at the Telehealth Center of the hospital, and was conducted by the Taiwan ELEctroHEALTH study group (TELEHEALTH study group). Patients aged 20 years or over receiving the telehealth program at our telehealth center were enrolled as the case group. The control group included subjects who visited our cardiovascular center during the same period but did not participate in the telehealth care program (received usual care only), and were matched for age, gender, and Charlson comorbidity index. Data on sex, gender, and diagnosis were obtained from the electronic database of our center. There were 7742 patients who visited our cardiovascular center during this period. After matching age, gender, and Charlson comorbidity index, 604 case patients and 1208 controls were selected. Follow-up data and medical costs of these subjects were then obtained from the electronic database. After excluding subjects with incomplete follow-up and cost data, a total of 576 cases and 1178 controls were finally enrolled in this study.

### Telehealth Care Program

**Figure 1:**
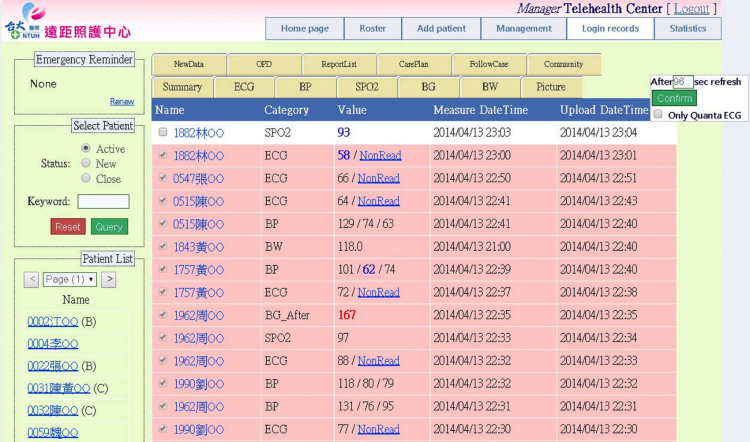
Screenshot of telehealth platform. All biometric and clinical data of patients receiving telehealth care can be assessed from this Internet-based database. Abnormal value marked in red.

The fourth-generation telehealth program at our telehealth center is a synchronized, structured, and integrated remote management program of chronic diseases [15]; it is an Internet-based system. Briefly, this telehealth program provided four major components. The first component was real-time transmission of biometric data from the patients to the telehealth center. The biometric data included single-lead electrocardiography, blood pressure, heart rate, oximetry, and glucometry (in patients with diabetes and those with impaired fasting glucose and impaired glucose tolerance). These biometric data were transmitted via the Internet and stored in the electronic health record system at our hospital. The data was processed immediately after transmission by the nurse case manager. Second, there were daily and on-demand telephone interviews between the telehealth care team and the patients for communication and health promotion. Third, full-time nurse case managers and cardiologists were in charge of care 24 hours a day. The nurse case managers reviewed the clinical and biometric data immediately and were empowered to adjust the dosage of medications or to stop a medication with potentially harmful side effects after consulting physicians. A screenshot of our telehealth platform is shown in Figure 1. The clinical information was relayed to the cardiology specialist who made the final judgment and suggestions regarding care. Fourth, the long-term medication and monitoring plan were discussed with the patients’ primary care physician after acute episodes. This telehealth program bridged between institute care and home care on an individualized approach, emphasized the prevention and early detection of clinical deterioration, and then managed the patient at the outpatient department (OPD) or emergency department (ED), rather than by hospitalization. The service and characteristics of this telehealth program did not change during the study period. All clinical information and biometric data were provided to the primary care physician at the regular OPD visit. Additional visits to the OPD were encouraged if adjustments to medications were frequently required or if inadequate control of symptoms was noted by the nurse case manager. An ED visit was suggested if acute deterioration of a chronic condition was suspected. The clinical information was relayed to the ED before the patient arrived at the ED. The decision to hospitalize was determined by primary care physicians and/or ED physicians.

### Usual Care

Patients in the control group received usual care provided by the cardiovascular center according to updated guidelines, including, but not limited to, the American Heart Association’s guidelines on lifestyle management to reduce cardiovascular risk, guidelines for the management of stable ischemic heart disease, and the American Diabetes Association’s guidelines for the management of diabetes. Patients in the control group received routine OPD visits to primary care physicians. ED visits were determined by patients and/or caregivers. The decisions for hospitalization were determined by primary care physicians and/or ED physicians. There was no contact between telehealth center and patients receiving usual care.

### Data Collection

All of the demographic, hospitalization, and payment data were collected from the electronic database of the hospital. The diagnosis of chronic diseases was also based on the electronic database. A discharge diagnosis was recorded if the outpatient and discharge diagnoses were different.

### Outcomes

The primary outcomes were hospitalization, length of hospitalization, and ED visits adjusted by the follow-up period (month). Data on hospitalization, length of stay, and ED visits were collected using an administrative billing system.

### Costs

#### Medical Costs

Taiwan launched a single-payer National Health Insurance program in 1995, which covers most of the medical costs from primary care to hospitalization. This study used a third-party payer perspective and included only direct medical costs in the cost analysis, including: medication, pharmacological service, examinations, diagnostic tests, physician visits, operations, anesthesia, blood product, ward, nursing, and specialized equipment during visits to the OPD and ED, and hospitalization. Self-paid medical costs (not covered by National Health Insurance program) were also included. Costs were adjusted to the US$ average exchange rate for 2012.

#### Telehealth Care Program Costs (Intervention Costs)

The total intervention costs of the telehealth care program included direct costs (in-house staff costs, contract costs, and fees to other organizations) and indirect costs (marketing, business development, and administrative costs). The costs for the telehealth equipment were included in the fees to other organizations. The intervention cost per patient-month was calculated by the total intervention costs in 2011 and 2012 divided by the active participants and duration (months).

#### Total Costs

The total costs were defined as the sum of medical costs and intervention costs in the telehealth group; the total costs were the same as medical costs in the control group.

### Cost-Effectiveness

Cost-effectiveness was evaluated by the cost saved for each hospitalization that was avoided and the cost per hospitalization day that was avoided. Uncertainty was calculated by the bootstrap method and plotted in a cost-effectiveness plane.

### Statistical Analysis

Discrete data are expressed as count and percentages. Continuous data are presented as mean and standard deviation (SD) or median and interquartile range (IQR) for data with normal or skewed distribution, respectively. The chi-square test was used to compare categorical data. The Student’s *t* test or Mann-Whitney test was applied to compare continuous unpaired data with normal or skewed distribution, respectively. A Wilcoxon rank-sum test was applied to compare the outcome data and costs, which were not normally distributed.

Linear regression models for the number of ED visits, hospitalizations, hospitalization days, and number of intensive care unit admissions were developed with age, sex, participation in the telehealth care program, and Charlson comorbidity index as the independent variables. To evaluate the variables that were significantly associated with cost, a two-part gamma model was adapted. First, logistic regression analysis was performed to evaluate the probability of the cost of the clinical factors being greater than zero. The significant variables were then entered into the second part, for which we use a generalized linear model (GLM) with gamma distribution and logarithmic link function to evaluate the variables in the patients with costs larger than zero.

The time to first ED visit or hospitalization-free survival was estimated according to the Kaplan-Meier method. The effect of participation in the telehealth program was estimated using a Cox proportional hazards model. Repeat hospitalizations were examined using Cox regression analysis for recurrent events, accounting for the possibility of multiple readmissions occurring over the follow-up period.

Cost-effectiveness was measured by:

(cost_case_−cost_control_) / (effect_case_−effect_control_)

The incremental cost-effectiveness ratio (ICER) provided the payments per hospital episode averted or per hospital day averted. Non-parametric bootstraps were used to simulate 5000 ICERs that were plotted on a cost-effectiveness plane. Each simulated ICER fell into one of the four quadrants of the ICER plane (increased or decreased cost vs better or worse health result). Strong dominance applied when the telehealth program was both more effective and less costly than usual care.

Because there was difference in the follow-up duration in two study groups, we performed sensitivity analyses by different follow-up durations. We repeated the analyses for the comparisons of clinical events, Cox regression for time-to-first admission free survival, comparisons of medical costs, and cost-effective plane by the different follow-up durations (<3 months, <6 months, <1 year, <2 years, 3 months-1year, 1-2 years, and without adjustment for duration).

A *P* value of less than .05 was considered to be statistically significant. Stata/SE 11.0 for Windows (StataCorp LP, College Station, TX, USA) was used for all statistical analyses.

## Results

### Descriptive Statistics

A total of 1754 patients (576 in the telehealth group and 1178 in the control group) were enrolled in this study. The average age was 64.5 (SD 16.0) years; 61.17% (1073/1754) of the subjects were male. The diagnosis of chronic CVD included hypertension (79.99%, 1403/1754), heart failure (16.99%, 298/1754), stroke (10.32%, 181/1754), myocardial infarction (7.13%, 125/1754), and peripheral artery diseases (5.19%, 91/1754). The mean Charlson comorbidity index was 1.26 (IQR 0-2). At baseline, age, sex, and Charlson comorbidity index were matched between the two groups (Table 1). There were slightly more patients with heart failure, stroke, dementia, chronic obstructive pulmonary disease, diabetes, and peptic ulcer disease in the telehealth group (Table 1). There was no difference in hemoglobin A1c level between two study groups. There was no difference in the duration of education between two study groups. The median follow-up time was 694 days (IQR 338-1163). The follow-up time in the control group (879 days, IQR 334-1190) was longer than that in the telehealth group (572 days, IQR 349-809). Because of the different follow-up times, the costs and events were divided by the follow-up time (months) in the subsequent analysis.

**Table 1:** Baseline characteristics.

Characteristic		Cases	Controls	*P* value
Patients, n		576	1178	
Age, year, mean (SD)		64.6 (16.3)	64.5 (16.1)	.8
Sex (male), n (%)		356 (61.81%)	717 (60.87%)	.7
Systolic blood pressure, mmHg, mean (SD)		124.3 (20)	123.3 (27)	.5
Diastolic blood pressure, mmHg, mean (SD)		70.4 (12.6)	70.6 (16.4)	.8
**Comorbidity**					
	Charlson comorbidity index	1.35 (1.65)	1.21 (1.52)	.07
	Myocardial infarction, n (%)	44 (7.64%)	81 (6.88%)	.5
	Heart failure, n (%)	112 (19.44%)	186 (15.79%)	.05
	Peripheral artery disease, n (%)	61 (10.59%)	30 (2.55%)	.9
	Stroke, n (%)	71 (12.33%)	110 (9.34%)	.05
	Dementia, n (%)	13 (2.26%)	10 (0.85%)	.01
	Chronic obstructive pulmonary disease, n (%)	49 (8.51%)	67 (5.69%)	.02
	Diabetes, n (%)	165 (28.65%)	273 (23.17%)	.01
	Peptic ulcer disease, n (%)	28 (4.86%)	34 (2.89%)	.03
	Chronic kidney disease, n (%)	57 (9.90%)	117 (9.93%)	.9
	Chronic liver disease, n (%)	15 (2.60%)	26 (2.21%)	.6
	Malignancy, n (%)	44 (7.64%)	120 (10.19%)	.08
**Hemoglobin A1c, %, mean (SD)**					
	Overall	6.6 (1.4)	6.7 (1.5)	.15
	With diabetes	7.2 (1.6)	4.7 (1.5)	.33
	With ICU^a^ admission	6.8 (1.5)	6.9 (1.7)	.65
**Duration of education, n (%)** ^b^				.09
	<9 years	102 (31.58%)	259 (47.01%)	
	9-16 years	200 (61.92%)	266 (48.28%)	
	>16 years	21 (6.50%)	26 (4.72%)	
**Medications, n (%)**				
	Anti-hypertension	475 (82.47%)	932 (79.11%)	.1
	Oral-anti-diabetes	93 (16.15%)	157 (13.33%)	.1
	Insulin	47 (8.16%)	47 (3.99%)	<.001
	Statin	74 (12.85%)	120 (10.19%)	.1
	Aspirin	117 (20.31%)	249 (21.14%)	.7
	Clopidogrel	128 (22.22%)	238 (20.20%)	.3

^a^ICU: intensive care unit.

^b^Not all patients provided their education information—data was obtained for 874 participants (323 in the telehealth program and 551 in the control group).

### Outcomes

There were significantly fewer ED visits, hospitalizations, hospitalization days, and intensive care unit admissions per month in the telehealth group compared to the control group (Table 2). The hospitalization-free survival was significantly longer in the telehealth group (*P*=.01, log-rank test). In the Cox regression analysis, age (hazard ratio [HR] 1.01, *P*<.001), telehealth (HR 0.76, *P*=.001), and Charlson comorbidity index (HR 1.23, *P*<.001) were independent predictors for re-hospitalization (Table 3). The ED visit-free survival was not significantly longer in the telehealth group (*P*=.08, log-rank test). In the Cox regression analysis, only age (HR 1.01, *P*<.001) and Charlson comorbidity index (HR 1.3, *P*<.001) were independent predictors for an ED visit. Repeated events Cox regression analysis also demonstrated significantly longer hospitalization-free survival for the telehealth group. Age (HR 1.01, *P*=.013), telehealth (HR 0.5, *P*=.001), and Charlson comorbidity index (HR 1.41, *P*<.001) were independent predictors for repeated hospitalizations (Table 3).

**Table 2:** Clinical events, adjusted by follow-up months.

Events	Cases (n=576)	Controls (n=1178)	*P* value
	mean (SD)		
Follow-up months	20.4 (11.4)	25.8 (14.5)	<.001
ED^a^ visits	0.06 (0.13)	0.09 (0.23)	<.001
Hospitalizations	0.05 (0.12)	0.11 (0.21)	<.001
Hospitalization days	0.77 (2.78)	1.4 (3.6)	<.001
ICU^b^ admissions	0.01 (0.07)	0.04 (0.14)	<.001
OPD^c^ visits	1.57 (1.12)	1.66 (1.78)	.75

^a^ED: emergency department

^b^ICU: intensive care unit

^c^OPD: outpatient department

**Table 3:** Multivariate Cox regression analysis for event-free survival.

	Time to first hospitalization		Time to first emergency department visit		Hospitalization, multiple event	
	Hazard ratio	*P*	Hazard ratio	*P*	Hazard ratio	*P*
Age	1.01 (1.01-1.02)	<.001	1.01 (1.0-1.01)	<.001	1.01 (1.0-1.02)	.013
Sex	1.11 (0.97-1.29)	.13	1.01 (0.86-1.19)	.9	0.94 (0.69-1.29)	.71
Telehealth	0.76 (0.65-0.89)	.001	1.11 (0.94-1.35)	.19	0.5 (0.34-0.74)	.001
Charlson comorbidity index	1.23 (1.19-1.28)	<.001	1.3 (1.25-1.35)	<.001	1.41 (1.32-1.52)	<.001

### Costs

The monthly intervention costs in the telehealth group were US$224.80 per patient (Table 4). The personnel costs comprised 77.97% (27,348.50/35,075.50) of the intervention costs. The average medical costs were US$587.60 (SD 1497.80) per month in the telehealth group and US$1163.60 (SD 3036.60) per month in the control group (*P*=.02; Table 5). Generalized linear model (GLM) analysis revealed that telehealth (OR 0.4, 95% CI 0.32-0.55, *P*<.001), heart failure (OR 1.56, 95% CI 1.11-2.19, *P*=.001), and cancer (OR 1.86, 95% CI 1.23-2.8, *P*<.001) were significantly associated with the total costs (all medical + intervention costs; Table 6). Hospitalization costs accounted for the largest portion of the total costs and were significantly higher in the control group (mean US$878.20 SD 2697.20 per month) compared with the telehealth group (mean US$386.30 SD 1424.30 per month; Table 5). GLM analysis also revealed that only telehealth (OR 0.67, 95% CI 0.46-0.95, *P*=.009) was significantly associated with the hospitalization costs (Table 6). The OPD costs were also higher in the control group (mean US$248.20 SD 984.60 per month) compared with the telehealth group (mean US$180.40 SD 248.20 per month; Table 5). GLM analysis further revealed that age (OR 1.01, 95% CI 1.0-1.01, *P*=.01), telehealth (OR 0.72, 95% CI 0.57-0.9, *P*=.05), heart failure (OR 1.61, 95% CI 1.2-2.17, *P*=.002), and liver cirrhosis (OR 4, 95% CI 2.0-8.1, *P*<.001) were significantly associated with the OPD costs. The ED costs were higher in the control group (mean US$20.90 SD 66.60 per month) compared with the telehealth group (mean US$37.30 SD 126.20 per month; Table 5). GLM analysis revealed that age (OR 1.02, 95% CI 1.01-1.03, *P*<.001), heart failure (OR 1.47, 95% CI 1.01-2.12, *P*=.05), and cancer (OR 2.3, 95% CI 1.48-3.6, *P*<.001) were significantly associated with the ED cost.

**Table 4:** Intervention cost (2011-2012).

Cost category	Amount (US$/month)
In-house staff	$27,348.50
Contract costs/Fees to other organizations	$5213.70
Total direct costs	$32,562.20
Marketing and business development	$15.50
Selling, general and administrative	$2451.20
Other expenses	$46.70
Total intervention cost	$35,075.50
Total intervention cost per patient	$224.80

**Table 5:** Medical cost (US$ per patient/month).

Medical costs		Case	Control	*P* value
		mean (SD)	mean (SD)	
**By clinical setting**				
	Emergency department costs	$20.90 (66.60)	$37.30 (126.20)	<.001
	Hospitalization costs	$386.30 (1424.30)	$878.20 (2697.20)	<.001
	Outpatient clinic visit costs	$180.40 (278.60)	$248.20 (984.60)	.06
	Total medical costs	$587.60 (1497.80)	$1163.60 (3036.60)	<.001
	Total health care costs	$812.40 (1497.80)	$1163.00 (3036.60)	<.001
**By items**				
	Laboratory examinations	$66.10 (171.10)	$120.2 (270.90)	<.001
	Imaging	$20.00 (56.20)	$56.40 (150.10)	<.001
	Medication	$130.00 (304.00)	$226.60 (864.50)	.009
	Other treatment and management	$56.10 (286.60)	$81.30 (315.00)	.11
	Physician visit	$16.10 (65.20)	$26.40 (69.40)	.003
	Nursing	$42.60 (224.30)	$69.40 (244.60)	.03
	General ward	$51.90 (240.00)	$59.70 (212.40)	.49
	ICU^a^ ward	$19.20 (135.70)	$30.30 (146.10)	.13

^a^ICU: intensive care unit.

**Table 6:** Generalized linear models for costs.

Costs/Factors	Outpatient department		Emergency department		Hospitalization		Total (medical + intervention)	
	OR	*P*	OR	*P*	OR	*P*	OR	*P*
Age	1.01 (1.0-1.01)	.01	1.02 (1.01-1.03)	<.001	1.00 (0.99-1.01)	.33	1.0 (0.99-1.01)	.7
Telehealth	0.72 (0.57-0.9)	.05	0.72 (0.51-1.01)	.06	0.67 (0.46-0.95)	.009	0.4 (0.32-0.55)	<.001
Heart failure	1.61 (1.2-2.17)	.002	1.47 (1.01-2.12)	.05	-	-	1.56 (1.11-2.19)	.009
Diabetes	1.32 (1.02-1.7)	.03	-	-	-	-		
Liver cirrhosis	4.0 (2.0-8.1)	<.001	-	-	-	-	-	-
Cancer	-	-	2.3 (1.48-3.6)	<.001	1.48 (0.97-2.28)	.07	1.86 (1.23-2.8)	.003

### Cost-Effectiveness

**Figure 2:**
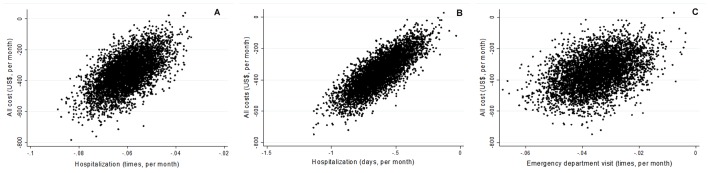
Cost-effectiveness planes for hospitalization times, hospitalization days, and emergency department visits averted.

Figure 2 shows the 5000 bootstrapped replicates of incremental costs versus hospitalizations, hospitalization days averted, and ED visits averted on a cost-effectiveness plane. Because of the different follow-up times, the cost, number of hospitalizations, and hospitalization days were divided by the follow-up months. In this bootstrap analysis, 99.9% of the 5000 replicates were in the cost-saving quadrant in all three analyses, which indicated that the telehealth program was a dominant strategy.

### Sensitivity Analyses

The sensitivity analyses by different follow-up durations revealed consistent results. There were no differences in age, sex, and Charlson comorbidity score in the two study groups by different follow-up durations. The clinical events were significantly less in the telehealth group over different follow-up durations. In the Cox regression analyses for time to first admission-free survival, the telehealth was a significant protective factor over different follow-up durations. The total medical costs were significantly lower in the telehealth group over different follow-up durations. In the cost-effectiveness analyses, the 1000-times bootstrap revealed that telehealth was a cost-saving strategy (99.9% of the simulations were in the cost-saving quadrant over different follow-up durations, except for the duration of 1-2 years where 96.6% of the simulations were in the cost-saving quadrant).

## Discussion

### Principal Results

The results of the current study revealed that our fourth-generation telehealth program was associated with lower total costs, lower rate of hospitalizations, and shorter hospitalization length of stay in patients with chronic CVD during the 2-year follow-up period. The total costs of US$811 per month (medical costs: US$587, intervention costs: US$224) in the telehealth group were less than the total costs of US$1163 (medical costs only) per month in the control group. In multivariate analyses, the telehealth program was an independent predictor for a longer time to first hospitalization and repeated hospitalization-free survival. In the GLM analysis, the telehealth program was independently associated with fewer OPD visits and lower hospitalization medical costs and total health care costs (medical + intervention cost). Sensitivity analysis revealed that the telehealth program was a cost-saving strategy from the health care system perspective.

This cost-saving conclusion was consistent over different endpoints, including hospitalization times and durations. Our intervention costs at US$224.80 per month (or US$2697 per year) were reasonable compared to those reported in two recent telehealth studies: US$3010 per year in a UK study and US$2177 per year in a US study [11,12]. Our synchronous telehealth program adopted a strategy to manage patients in OPD or ED without delay to avoid hospitalization, if an acute event was detected. This strategy showed a 38% reduction in hospitalizations without significant increase in OPD or ED visits in the telehealth group (Table 2). The non-significantly less ED visit-free survival in the telehealth group (Figure 1B) did not translate into higher costs or more hospitalizations. Based on these advantages in costs and event reduction, our telehealth program was demonstrated to be cost-effective.

### Comparison With Prior Work

Until now, research on telehealth programs in chronic diseases management has had mixed results [13,16]. Telehealth care has been shown to reduce hospitalizations in patients with chronic conditions such as asthma [6], chronic obstructive pulmonary disease [7], and heart failure [8,9]. Data also shows that telehealth care can achieve better blood pressure [17,18] and glycemic control [19]. Two large randomized controlled trials published recently, however, questioned the benefits and cost-effectiveness of telemedicine in patients with chronic diseases [11,12]. The first trial enrolled 205 elderly adults aged 60 years or above, with multiple illnesses and a higher risk of hospitalization [11]. Half of the participants (51.2%) had chronic CVD. The results showed a neutral effect of asynchronous telemonitoring on the composite endpoint of hospitalization and ED visits in 12 months (63.7% vs 57.3%, *P*=.35). A higher mortality rate was reported in the telemonitoring group compared to the control group unexpectedly (14.7% vs 3.9%, *P*=.008). Although the study was carefully designed and conducted, only 40% of the subjects screened were enrolled in the trial. This low rate of enrollment may limit its generalizability. Another factor that may limit its generalizability is the asynchronous telemonitoring used in this trial.

The second randomized controlled trial (Whole System Demonstrator trial [WSD]) enrolled 3230 people with heart failure (37.8%), chronic obstructive pulmonary disease (33.5%), or diabetes (28.7%) in the United Kingdom. The results showed the hospitalization rate (OR 0.85, *P*=.017), length of hospital stay, and mortality (4.6% vs 8.3%, *P*<.001) were reduced in the telehealth group [20]. These reductions in hospitalization and length of hospital stay in the telehealth group were similar to our results. A cost-effectiveness analysis of this trial included 965 of the original 3230 subjects, among them 36.4% had heart failure. The result derived an ICER of £92,000 per QALY gained. This value was considered not to be cost-effective compared with the current threshold of willingness to pay [12]. The medical costs in the telehealth group were lower than those in the usual care group (not including the intervention costs). This indicates that the cost of intervention is the major determinant for the cost-effectiveness of a telehealth care program. The intervention costs in our study were lower than those in the WSD trial. This difference in intervention costs may account for the difference in cost-effectiveness between our study and the WSD trial.

The causes of these inconsistent results between different trials are not totally clear. Our data provide three potential implications for the causes. First, our data imply that the level of care provided by a telehealth program affects its efficacy. Different telehealth programs should not be compared directly without analyzing their basic structure. An effective telehealth program relies on the premise that routinely monitoring biometrics and symptoms will facilitate early detection of clinical deterioration and trigger timely intervention [21]. To achieve this goal, our fourth-generation telehealth program has the ability to detect deterioration early by synchronous data analyses and to initiate timely intervention by round-the-clock presence of a physician. Our telehealth program implemented an electronic platform to integrate all the biometrics measured and to notify the nurse case managers immediately if abnormal values were received. The nurse case managers could respond more rapidly to abnormal biometric values, compared with the stored-and-forward system used in other trials [11,22]. The nurse case managers were trained to deliberately seek and track the symptoms and signs of early deterioration during the daily telephone interview. This strategy may detect early deterioration more efficiently in patients with a wide range of comorbidities, compared with the patients answering predetermined screening questions to automated systems used in other trials [11,22]. Prior research has shown that the benefit of a telehealth program in patients with heart failure can be lost after changing from a small-scale, nurse case manager-led program to a large-scale, automated monitoring system without one-to-one telephone interviews [22,23]. These results highlight the role of nurse case managers in a telehealth program. The round-the-clock presence of a physician for therapeutic decisions and the synchronous type of telehealth program adopted in our study were not formally tested in other trials. Although we did not directly compare our telehealth program with an earlier generation, our data imply that level of care provided by a telehealth program makes a difference in efficacy. Future research on telehealth programs should clearly address the level of care provided by the program. We suggest a synchronous telehealth program should be considered in a population with multiple comorbidities and high risk for acute deterioration, as the program implemented in our study.

The second implication of our data is that the composition of the total health care costs is crucial to the balance of cost-effectiveness in a telehealth program. The intervention costs accounted for 27% of the total cost in the telehealth group in our study; the personnel cost accounted for 78% of the intervention costs. This result indicates that one-fifth of the total health care cost of the patients receiving our telehealth program will pay for the personnel costs. The personnel costs may not change much if a specific level of care is determined. The medical costs, however, increased significantly with older age and more comorbid conditions, as demonstrated by our GLM analyses. Given a patient population with fewer comorbid conditions than the patients in our study, the expected annual medical costs would be much lower. If the medical costs are much lower than the intervention costs, the cost-saving feature of the telehealth program may not exist. The same problem will also be encountered in a patient population with a greater number of comorbid conditions and higher expected medical costs. Therefore, choosing the appropriate patient population would have a major influence on the balance of cost-effectiveness. According to our data and prior trials in telehealth programs [12] or disease management programs [24], the intervention costs should be less than US$2500-3000 per year to be cost-saving in a patient population with moderate chronic comorbidities. With advances in information technologies such as automatic data analysis algorithms, the personnel costs might be lowered in the future.

The third implication of our data is that the patient population that will benefit from a telehealth program extends to patients with chronic CVD and multiple chronic conditions. In CVD, telehealth programs have been tested mostly in patients with heart failure [14,22,23,25]. In our study, 24% of our patients had heart failure or myocardial infarction. The mean Charlson comorbidity index in our study was 1.26, which was lower than that reported in some of the recent research [11,12]. The overall severity in our study may be less than that in the studies in patients with heart failure. Our study provided the data of a telehealth program for a patient population with established CVD and multiple comorbidities but without advanced heart failure.

### Limitations

There are several limitations to this study. First, because this was not a randomized controlled study, confounding factors and bias may not have been detected. Although we matched the patients and controls by age, sex, and Charlson comorbidity index, each item of the Charlson comorbidity index was not completely matched. However, because the disease severity was higher in the telehealth group, we do not think that this artificially increased the benefits of telehealth care. Moreover, a protocol-driven use of resources did not exist in this study, making the costs more reflective of the real-world situation. Other confounding factors such as socioeconomic status were not fully detected in this study. Second, only direct medical costs and intervention costs were reported in our study; however, travel and time costs are two major direct non-health-related costs. These two types of cost are difficult to compare between different societies and health care systems, and therefore we did not include these two costs in our analysis. Third, the clinical outcomes were derived from the electronic billing and medical records of our hospital, and the patients who received care outside of our hospital were not recorded. Resources that were used but not billed may also have been overlooked when extracting data from our billing system. Fourth, long-term follow-up data were not available in our study, so the cost-effectiveness over a longer time frame is unknown. Finally, we did not measure the QALY in our study, although many cost-effectiveness studies have reported this. Although frequently used in health economic research, QALY is not without drawbacks [26]. One of the major problems is that the use of QALY rests on the assumption that all QALYs are of equivalent value in the perspective of society. However, this assumption is not necessarily true in all circumstances [27]. QALYs derived from different societies and cultural backgrounds may not be suitable for a direct comparison. Hence, we reported the cost per hospitalization avoided.

### Conclusions

Our data support that a fourth-generation telehealth program is associated with a reduction in the rate of hospitalizations, the length of hospital stay, and the accompanying medical costs in patients with chronic CVD and multiple comorbidities. The intervention costs of this new generation of telehealth program do not increase the total costs for patient care. Randomized trials should be considered in this new generation of telehealth program for the management of chronic CVD.

## Abbreviations

CVD: cardiovascular disease
ED: emergency department
GLM: generalized linear model
ICER: incremental cost-effectiveness ratio
IQR: interquartile range
OPD: outpatient department
QALY: quality-adjusted life year
TELEHEALTH: Taiwan ELEctroHEALTH study group
WSD: Whole System Demonstrator trial
